# Pulmonary Fibrosis Induced by CdSe Nanorods and the Therapy with Modified Procyanidinere

**DOI:** 10.3390/toxics10110673

**Published:** 2022-11-08

**Authors:** Zongkai Yue, Ruiren Zhou, Qingzhao Li, Shaohu Ouyang, Lu Liu, Qixing Zhou

**Affiliations:** 1 Laboratory of Environmental Protection in Water Transport Engineering, Tianjin Research Institute for Water Transport Engineering, Ministry of Transport of the People’s Republic of China, Tianjin 300456, China; 2 Ministry of Education Key Laboratory of Pollution Processes and Environmental Criteria/Tianjin Key Laboratory of Environmental Remediation and Pollution Control, College of Environmental Science and Engineering, Nankai University, Tianjin 300071, China; 3 Department of Biological and Agricultural Engineering, Texas A&M University, College Station, TX 77843-2117, USA; 4 Preventive Medicine Department and Department of Biological Science, Hebei United University, Tangshan 063000, China

**Keywords:** pulmonary fibrosis, CdSe nanorod, oral administration, nanotoxicology, procyanidinere therapy

## Abstract

The CdSe nanorod as a one-dimensional nanostructure has an excellent performance in many fields, such as healthcare, new energy, and environmental protection. Thus, it is crucial to investigate its potential adverse health effects prior to their wide exposure. The lung tissue would be the main target organ after CdSe nanorods enter living systems. Here, we showed that pulmonary instillation of CdSe nanorods could decrease the vitality of T-SOD and T-AOC in lung tissues of a rat, increase MDA and hydroxyproline levels and lipid peroxidation products, induce mitochondrial cristae breakage and vacuolization, cause inflammatory responses, and finally induce pulmonary fibrosis. The oral administration of modified procyanidinere could significantly increase the content of antioxidant enzymes, scavenge free radicals, reduce lipid peroxidation, and have protective effects on CdSe nanorods-induced pulmonary fibrosis. The benefit is not only in the early inflammatory stage but also in the later stages of the CdSe nanorods-induced pulmonary fibrosis.

## 1. Introduction

Due to their small scale of compositive units, considerable specific surface area, and high surface reactivity [1], nanomaterials exhibit unique physicochemical, mechanical, electrical, and thermal properties that can be applied in healthcare and medicine, electronic information, energy storage, and environmental protection [2,3,4]. Compared with their bulk counterparts, nanomaterials generally enter into living organisms more easily, in particular, lung tissues, and are more toxic [5,6,7]. They might enter into cells through free penetration or receptor-mediated endocytosis and actively interact with cellular components, such as lipids, proteins, and genomic DNA [8,9,10,11]. For example, a number of toxicological studies using rats have shown that exposure to nanomaterials such as carbon nanotubes, nickel, and TiO_2_ nanoparticles induces greater lung inflammatory potency and cytotoxic effects than larger-size particles at equivalent mass concentrations [12,13,14]. One-dimensional (1D) nanostructures have attracted much attention in the past decade owing to their unique optical and electrical properties, and they are good candidates as the building blocks of functional nanodevices such as field-effect transistors [15,16,17], photodetectors [18], light-emitting diodes [19], and photovoltaic devices [20]. CdSe nanoparticles have been widely applied in photoelectric conversion, biomedical imaging, and drug delivery [21,22]. CdSe nanorods as one-dimensional nanostructures have excellent performance in photoelectric conversion as they could provide a natural channel for directional electron transport, and the rods’ structure made the transfer resistance smaller and the transmission speed faster [23]. Just as many other nanomaterials [24], CdSe nanoparticles have been demonstrated to be toxic to mammalian cells and tissues [25,26,27].

To date, most nanomaterial toxicity studies have focused on quantum dots. There are many reports showing that lung tissue exposed to quantum dots might lead to reactive oxygen species (ROS) accumulation [28,29,30], inactivation of protein functions [31], and cause pulmonary fibrosis in lung tissues [32]. However, only a few studies have addressed the toxicity mechanisms of rod-like nanoparticles and the corresponding therapy recommendations [32,33,34]. Although there have been reports of cytotoxicity induced by some metallic nanoparticles [35], there is no comprehensive study of the toxicity of CdSe nanorods to lung tissues and lung cells. Accompanied with the application of CdSe nanorods, lung tissues would be the main target organ after CdSe nanorods enter into life systems or biological bodies. Through the respiratory tract route, CdSe nanorods would deposit in the bronchial epithelium, pulmonary interstitial, and alveolar walls. For a foreign matter in lung tissues, the defense and removal functions of macrophages could be activated, but it is difficult for macrophages to identify such a small granule. Their function is being weakened [36], So CdSe nanorods might be uptaken by cells, penetrate across the barrier into circulation, and migrate to the liver and other organs or tissues [37,38,39].

For many years, although many efforts have been made in fighting pulmonary fibrosis [40,41] and various drugs, such as anti-inflammatory [42], immunosuppressive [43], and anti-fibrotic agents [44], have been clinically used for the treatment of pulmonary fibrosis, there has been only very limited success case. As such, it is desirable to search for new therapeutic strategies or recommendations. Procyanidine (OPC) has a special molecular structure of biological flavonoids derived from grape seeds, and it has been internationally recognized as the most effective natural antioxidant, which can clean up free radicals in life systems or biological bodies [45]. Thus, it had been reported to exert antibacterial, antiviral, anticarcinogenic, antimutagenic, anti-inflammatory, antiallergic, and vasodilatory actions [45,46,47]. Most of its physiological benefits have been attributed to its antioxidant and free radical scavenging properties [48]. OPC exhibited dramatic scavenging ability towards biochemically generated superoxide anions, hydroxyl, and peroxyl radicals [49,50,51]. Furthermore, OPC has been shown to modulate the expression of apoptotic-related genes, reduce the generation of free radicals, and increase the activity of antioxidant enzymes in various types of animal tissues and cells, implying that it is a promising cytoprotective agent against a range of exogenous toxic stimuli [52].

In this work, we investigated the potential effects of synthesized CdSe nanorods on the lung tissues of SD rats and A549 cells for the first time, examined the effects of oxidative stress, and explored possible toxicity mechanisms of CdSe nanorods in the lung tissue of rats. Considering the antioxidant property of OPC, we explored its use in vivo and in vitro against adverse effects caused by CdSe nanorods.

## 2. Materials and Methods

### 2.1. Reagents

All chemical reagents were purchased from the Beijing Chemical Reagent Ltd., Beijing, China, and used without further purification. OPC purchased from the Beijing Chemical is a standardized water–ethanol extract from grape seeds. The extract was supplied in the form of standardized 95% and modified using physiological saline before use. Total antioxidant capacity (T-AOC), total superoxide dismutase (T-SOD), and malondialdehyde (MDA) assay kits were purchased from the Nanjing Jiancheng Bioengineering Institute, China.

### 2.2. Preparation and Characterization of CdSe Nanoparticles

For the synthesis of CdSe nanorods, 0.266 g Cd(CH_3_COO)_2_·2H_2_O and 0.345 g sodium selenite were added into 50 mL capacity Teflon-lined stainless autoclave, followed by the addition of 20 mL distilled water and 20 mL ethanediamine. The mixture was stirred using magnetic stirrer to a homogeneous system. The autoclave was placed in an oven at 150 °C for 12 h. After completion of the dual time, autoclave is allowed to cool to room temperature. The black product was collected and washed with distilled water and ethanol 6 times respectively and dried at room temperature for further use. The general morphology of the products was characterized by transmission electron microscopy. The crystal structure and composition of the sample were characterized by powder X-ray diffraction (XRD, D/MAX-2500, JAPAN SCIENCE) with Cu Kα radiation (λ = 1.54056 Å). CdSe nanorods were dispersed in saline and RPMI medium with a final concentration is 20 μg/mL respectively, and ultrasonic vibration was fully carried out to ensure their dispersion. The solution was allowed to stand for 24 h, dynamic light scattering (DLS) and ζ potential characterization of CdSe nanorods, which dispersed in saline and DMEM (0 h and 24 h, respectively) were determined using a Zetasizer (ZS90, Malvern Instruments, Worcestershire, UK.).

### 2.3. Animal Administration and Sampling

The main purpose was to estimate the toxicity to respiratory systems with CdSe nanorods and not concerned about gender differences. Hence, adult specific-pathogen-free (SPF) Sprague-Dawley (SD) male rats were employed in this study, SD rats (8–9 weeks old at the start of the study, 200–250 g/rat, the Center for Experimental Animals of Hebei United University) were used. For housing of animals, plastic cages filled with hardwood bedding were placed within an air-conditioned (23 ± 2 °C) animal room and with relative humidity ranging from 30 to 70%. A 12 h light/dark cycle was maintained throughout the study, except during the exposure, with free access to standard laboratory rats’ diet and tap water. After acclimation for 1 week, 72 male SD rats were randomly divided by weight into 9 groups (control groups, CdSe groups, and OPC groups administrate for 30, 60, and 90 days, respectively) with eight rats per group. Rats of control groups received pulmonary instillation of saline based on body weight. Rats of CdSe groups were administered by pulmonary instillation of 15 mg/kg CdSe nanorods, which dispersed with saline water per week. Rats of OPC groups were administered by gavage of a dose of 200 mg/kg OPC per day through the whole experiment process, meanwhile, were administered by pulmonary instillation of 15 mg/kg CdSe nanorods, which dispersed with saline water per week, respectively. Rats of corresponding groups were weighed and sacrificed on days 30, 60, and 90 (for schematic representation, see Figure S1). Parts of fresh lung samples were quickly collected and fixed in 4% paraformaldehyde or 2.5% glutaraldehyde solution, and the other lung samples were stored at −80 °C for further tests. A part of the right lung tissues of all rats in each group were selected for biochemical analysis. At the same time, a part of the left lung tissues of all rats in each group were used for histopathology staining and evaluation.

### 2.4. Cell Culture

The human lung carcinoma A549 cell line was obtained from the School of Public Health, Hebei United university. A549 cells were cultured in RPMI-1640 medium containing 100 μg/mL of penicillin, 100 μg/mL of streptomycin, and 10% heat-inactivated fetal bovine serum (FBS) at 37 °C in a humidified atmosphere of 5% CO_2_. Upon reaching 80–90% confluence, the cells were trypsinized, harvested, and seeded into a new cell culture dish. The control group was treated with vehicle and RPMI-1640 medium, the CdSe group was treated with CdSe nanorods (at a final concentration is 20 μg/mL), vehicle and RPMI-1640 medium, while the OPC group was treated with CdSe nanorods and OPC (at a final concentration is 20 and 40 μg/mL, respectively), vehicle and RPMI-1640 medium, 3 groups above were treated for 24 h. A549 cells were used to determine the oxidative damage effects of CdSe nanorods and the protective effect of OPC.

### 2.5. Determination of Oxidation Damages in Lung Tissues and A549 Cells

About 1.0 g of frozen lung tissues in 9 mL of homogenization buffers (0.9% sodium chloride) were homogenized on ice by a homogenizer (VCX130, Sonics & Materials, Inc., Newtown, CT, USA), working for 5 s each time, pause for 10 s, repeatedly for 4 times. The homogenate was centrifuged at 4000 rpm for 15 min at 4 °C and the supernatant was used for analysis. The T-SOD, T-AOC, and MDA dynamics were measured using T-SOD, T-AOC, and MDA assay kits for A549 cells and homogenate of lung tissues according to the instruction, respectively.

### 2.6. Histopathological Analysis of Lung Tissues

Fixed lung tissues (4% paraformaldehyde) were embedded in paraffin and then cut into 4 μm slices, which were mounted on glass microscope slides. The mounted sections were stained with hematoxylin-eosin (H&E) and examined by light microscopy. For the Masson’s trichrome staining, in strict accordance with the instructions of the Masson staining kit (D026-1, Nanjing Jiancheng Co., Ltd., Nanjing, China), sealed and finally examined by a light microscopy. Cell identification, cell morphology description, and alveolar wall thickness were evaluated by professional teachers and certified physicians.

### 2.7. Transmission Electron Microscopy

Animals designated for transmission electron microscopic examination (TEM) were sacrificed, opening the thorax of animals followed by perfusion of 5% buffered glutardialdehyde (GAH) as fixation solution. The tissue samples of the lungs were refixed with 2% buffered osmium tetraoxide aqueous solution. The fixed tissue embedded in epoxy resin, from appropriate locations ultrathin sections (50 nm) were obtained and observed by a Hitachi H-7650 transmission electron microscope (Tokyo, Japan) operated at 80 kV.

### 2.8. Determination of Hydroxyproline Level in Lung Tissues

On days 30, 60, and 90 after CdSe and OPC administration, tissue samples were weighed and cut into pieces, and 1 mL HCl (6 M) was added into the grinding test tube, which was capped and hydrolyzed in boiling water for 5 h. The pH of the solution was adjusted between 6.0 and 6.8. A total of 1 mL diluted solution supernatant was obtained for determination. This experiment included the following three groups: blank, standard, and detected sample tubes. Distilled water (1 mL) was added to the supernatant, as well as 5 μg/mL standard solution and analysis solution. The supernatant was analyzed (A030-3-1, acid hydrolysis, Nanjing Jiancheng Co., Ltd., Nanjing, China) at 550 nm with a spectrophotometer. The blank tube solution was used as zero control [53].

### 2.9. Statistical Evaluation

Data are expressed as mean ± SD (for histology). For in vitro methods, 6 independent experiments were performed, unless specified otherwise. For in vivo techniques, 4 (histopathology and transmission electron microscopy) rats per treatment group are used. Data were analyzed using SPSS version 15 for windows. Treatment-related differences were evaluated by one-way analysis of variance (ANOVA) or the nonparametric Mann-Whitney U-test (Bio-plex assay and histopathological scoring). Multiple comparisons were assessed by the Anova post hoc analysis according to Tukey’s method or the LSD method.

## 3. Results

### 3.1. Effects of CdSe Nanorods in Lung Tissues after Pulmonary Instillation

TEM and XRD images of the as-prepared CdSe nanorods are shown in Figure S2. Black-colored CdSe nanorods (NRs) have diameters ranging from 40 to 60 nm with lengths of 150–300 nm (Figure S2a and Table S1). The powder XRD pattern exhibited in Figure S2b corresponds to the hexagonal phase of CdSe (a = 4.299 Å, c = 7.01 Å, JCPDS: 08-0459). The hydrodynamic diameter of the synthesized CdSe nanorods in saline was also determined by DLS and the result is listed in Table S1.

**Figure 1:**
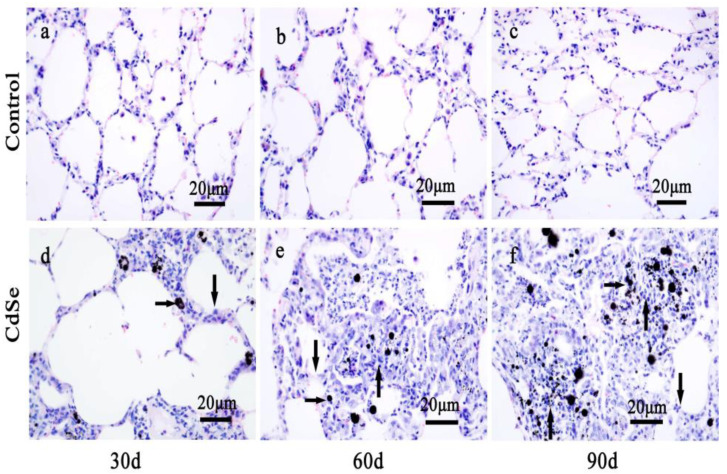
The alteration of lung tissue pathological morphology of SD rats induced by CdSe nanorods. The control groups that pulmonary instillation with saline for 30 days (**a**), 60 days (**b**), and 90 days (**c**). Pulmonary instillation CdSe nanorods for 30 days (**d**), 60 days (**e**), and 90 days (**f**). Blackspots are CdSe nanorods-positive sites (right arrows), the lung tissues from CdSe nanorods-exposed rats showed alveolar mucosa with swelling (down arrows), nodular histiocyte hyperplasia (up arrows), and injury. The above typical pictures are selected from the lung tissues of 8 rats in each group.

The histological changes of SD rat lung tissue sections with Hematoxylin-Eosin (H&E) stained were observed under light microscopy [54]. The representative images are presented in Figure 1. The H&E staining revealed that the lung structures in the control groups administrated with the physiological saline were found to be the normal morphology and alveolar walls composed of single epithelial cells. The normal alveolar cells with the equilibrium size are seen in the alveolar corner (Figure 1a). There was also slight interstitial inflammation found after pulmonary instillation of physiological saline for 60 and 90 days (Figure 1b,c), which appears as a normal phenomenon.

Compared with those in the control groups, the CdSe nanorod-treated groups produced significant adverse effects. Numerous black spots were observed under light microscopy. These black spots correspond to the CdSe nanorods-positive sites (right arrows in Figure 1d–f). The instillation of CdSe nanorods induced an extensive inflammatory response, including alveolar walls widening, alveolar mucosa with swelling, and congestion (down arrows in Figure 1d). As a result, inflammatory cell infiltration, and injury were observed in CdSe nanorod-treated SD rats on day 30 (Figure 1d). Followed by the pulmonary instillation of CdSe nanorods on days 60 and 90, black spots corresponding to the CdSe nanorod-positive sites were frequently observed under a microscope. Macrophages were also frequently observed in some of the alveolar walls. Inside the alveolar cavities, the thickness of the alveolar wall gradually thickens with time (down arrows in Figure 1e,f). Importantly, the part of the lung tissue that degenerate changed. The typical nodular histiocyte hyperplasia (up arrows in Figure 1e,f) [55], providing an encapsulation of the fibrous materials, also known for other particulate/fibrous materials [56] (quartz and asbestos), were obviously found on days 60 and 90 when compared with those for healthy lung tissues (the control groups).

### 3.2. Pulmonary Fibrosis Level in Lung Tissues

**Figure 2:**
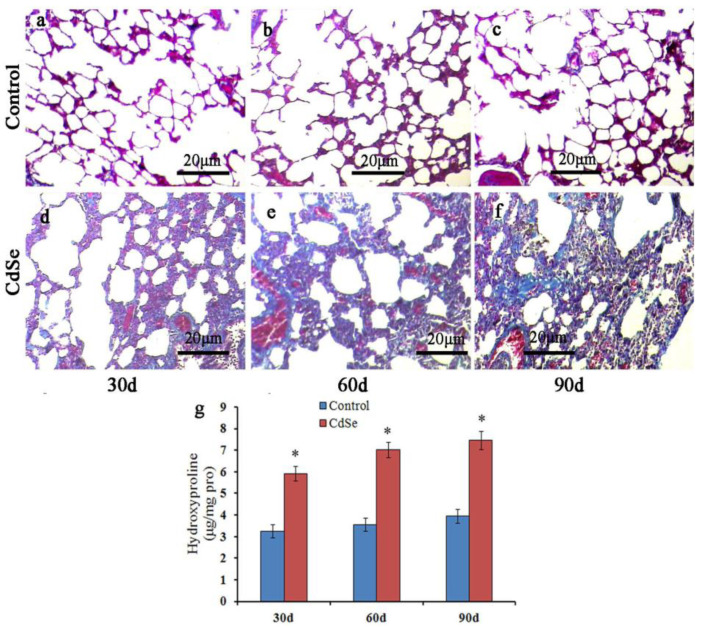
Masson trichrome staining to assess the pulmonary fibrosis. Compared with control groups (**a**–**c**), the collagen deposition increased significantly in lung interstitium at 30 (**d**), 60 (**e**), and 90 (**f**) days in CdSe nanorod-induced lung tissues. Detection of hydroxyproline content in lung tissues (**g**), similar to the changes in the collagen level, hydroxyproline level was also enhanced in CdSe nanorods-induced lungs on days 30, 60, and 90. Data are shown as mean ± SD (* *p* < 0.05, as compared with those in the normal control groups). The above typical pictures are selected from the lung tissues of 8 rats in each group.

In order to determine the degree of CdSe nanorods-induced pulmonary fibrosis, the Masson’s trichrome stain of lung tissue slices was evaluated. The representative results are depicted in Figure 2. The normal lung cells in the control groups were dyed a red color, while filament collagen fiber dyed a blue color, which is mingled in the normal lung cells, belongs to the normal phenomenon. Pulmonary fibrosis increased significantly in SD rats’ pulmonary system after the instillation of CdSe nanorods on days 30 and became more serious on days 60 and 90, along with collagen deposition, which also increased with the progression of instillation. In addition, the CdSe nanorod treatment significantly increased the hydroxyproline content of the lung compared with the control groups (Figure 2g). The hydroxyproline level is an indicator to measure collagen deposition in fibrosis [57]. We found that the hydroxyproline level significantly increased on days 30 and slightly increased on days 60 and 90 in the case of CdSe nanorods-treated lungs of rats compared with the control groups, which is according to the result obtained from the Masson’s trichrome stain. These results demonstrated that pulmonary instillation of CdSe nanorods could induce pulmonary fibrosis.

### 3.3. Oxidative Stress in Lung Tissues

**Figure 3:**
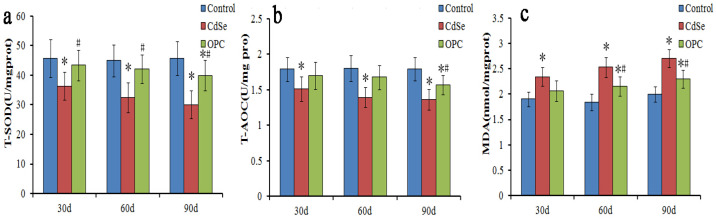
The bar graph showing SOD and T-AOC activity (**a**,**b**) and MDA content (**c**) in lung tissues of rats. They were measured using microplate reader represented as mean ± SD in the control groups, CdSe nanorods groups and OPC treatment groups for 30, 60, and 90 days (* *p* < 0.05, as compared with the normal control groups, # *p* < 0.05, as compared with the CdSe groups).

To evaluate oxidative stress caused by pulmonary instillation of CdSe nanorods, the activity of T-SOD and T-AOC, and MDA content levels in the lung tissues of a rat were measured (Figure 3). According to Figure 3, it was found that the activity of T-SOD and T-AOC in lung tissues of rats treated with CdSe was significantly lower with continual instillation than that in the control groups, while the MDA levels were significantly higher than those in the control groups. T-SOD levels dropped below control levels thus possibly allowing for unmetabolized SO to participate in the peroxide reaction. CdSe nanorods stimulated the lung tissue to produce abundant superoxide anion radicals and enhance the peroxide reaction.

### 3.4. OPC Therapy

**Figure 4:**
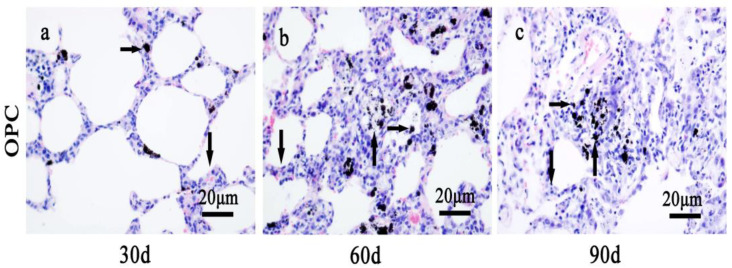
The alteration of lung tissue pathological morphology of SD rats after the repair strategy with OPC. (**a**) Oral administration of OPC for 30 days, (**b**) oral administration of OPC for 60 days, and (**c**) oral administration of OPC for 90 days. Black spots are CdSe nanorod-positive sites (right arrows), the lung tissues from OPC groups showed alveolar mucosa with slightly swelling (down arrows), relatively few nodular histiocyte hyperplasia (up arrows). The above typical pictures are selected from the lung tissues of eight rats in each group.

Lung fibrosis caused by pulmonary instillation of CdSe nanorods and modified OPC treatment in vivo mitigated the extent of inflammation (Figure 4). The histological morphological determination demonstrated that in gavages with OPC for 30, 60, and 90 days, along with alveolar walls widening, alveolar mucosa with swelling, inflammatory cell infiltration, and macrophages were seen in some of the alveolar walls compared with those in the control groups. However, the OPC treatment groups could significantly prevent the CdSe nanorods-induced inflammation and fibrosis [58] when compared with the CdSe nanorod groups for 30, 60, and 90 days, respectively. Lung tissues had less nodular histiocyte hyperplasia, lung fibrosis was significantly mitigated, the number of fibrocytes was also significantly decreased, and the thickness of alveolar walls was thicker compared with that in the control groups and thinner compared with that in the CdSe nanorod groups.

Similar to other toxicity indicators discussed about lung tissues, T-SOD, T-AOC, and MDA levels indicated a greater effect for the rats gavaged with the OPC solution (Figure 3). The activity of T-SOD and T-AOC had slightly decreased, and the MDA levels had slightly increased when compared with those in the control groups after being gavaged with the OPC solution. However, the activity of T-SOD and T-AOC in the OPC groups was significantly higher, and the MDA level was significantly lower when compared with that in the CdSe groups. The findings indicated that those gavaged with the OPC solution could prevent cytotoxicity mediated by free radicals and lipid peroxidation, and protect low-density lipoproteins from oxidation, while CdSe nanorods stimulated lung tissues to produce abundant superoxide anion radicals.

**Figure 5:**
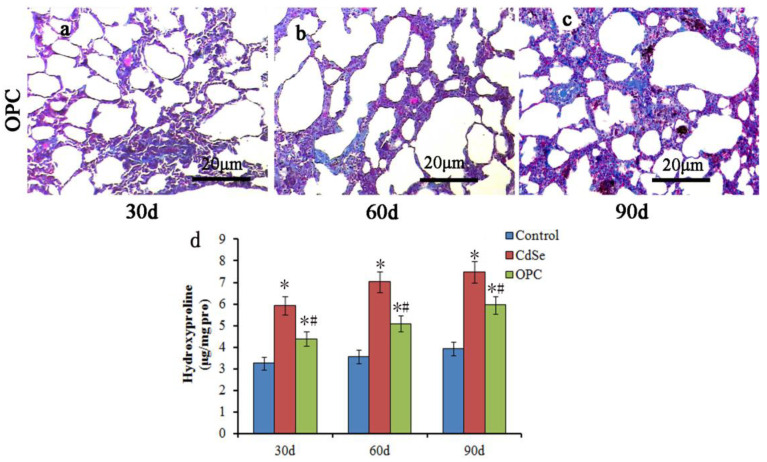
Masson trichrome stain in assessing the pulmonary fibrosis by the OPC effect. The collagen level decreased sharply in the OPC intervention groups compared with that in the CdSe groups at 30 (**a**), 60 (**b**), and 90 (**c**) days, respectively. OPC decreased the hydroxyproline levels effectively in the OPC-treated pulmonary fibrosis lung tissues when comparing with the CdSe groups (**d**). (* *p* < 0.05, as compared with the normal control groups, # *p* < 0.05, as compared with the CdSe groups). The above typical pictures are selected from the lung tissues of 8 rats in each group.

The Masson staining (Figure 5a–c) showed that the collagen deposition in the OPC group also gradually increased with the progression of pulmonary fibrosis when compared with the control groups, but the collagen level decreased sharply in the OPC intervention groups when compared with the CdSe groups, which is further supported by the hydroxyproline level and the collagen level analysis in the lungs (Figure 5d). Hydroxyproline levels decreased in the OPC-administrated lung tissues compared with those in the CdSe nanorods-treated lungs on days 30, 60, and 90, respectively. The hydroxyproline level of OPC-administrated lung tissues on days 90 (5.95 ± 0.43 μg/mg pro) is equivalent to the level of CdSe nanorod-treated lung tissues on days 30 (5.92 ± 0.41 μg/mg pro). The protective effect of oral OPC is obvious at the preliminary stage of pulmonary fibrosis induced by CdSe nanorods, and the observed effect is persistent with the progress of pulmonary fibrosis until 90 days.

### 3.5. Ultrastructure of Alveolar Macrophage and Oxidative Stress of A549 Cells

**Figure 6:**
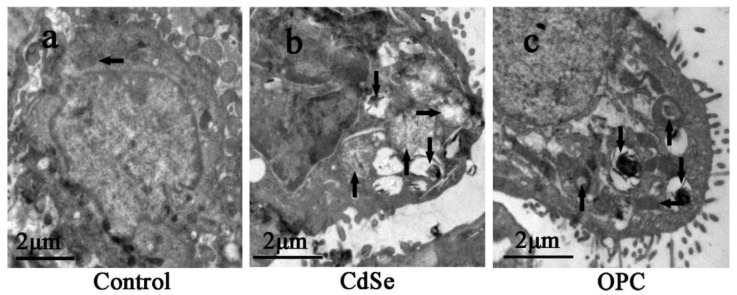
TEM images of ultrathin sections made with alveolar macrophage in the lung tissues. (**a**) the control group with normal mitochondria (left arrow); (**b**) the CdSe group, mitochondrial cristae broken, swelling (up arrows), and vacuolization (right arrows), numerous secondary lysosome containing aggregated CdSe nanorods (down arrows) were observed; (**c**) the OPC group, mitochondria became slightly swelling, less vacuolization (up arrows) were observed, secondary lysosome (down arrows) and normal mitochondria (left arrow) were also observed. The above typical pictures are selected from the lung tissues of eight rats in each group.

As the TEM images of ultrathin sections of alveolar macrophage in lung tissues, we observed that the control groups had normal mitochondria in alveolar macrophage. Numerous phagolysosomes in the CdSe groups showed broken mitochondrial cristae, swelling (up arrows in Figure 6b), vacuolization (right arrows in Figure 6b), and aggregated CdSe nanorods mainly located within secondary lysosomes (down arrows in Figure 6b) of alveolar macrophages. In the OPC groups, the part of mitochondria that was normal (left arrow in Figure 6c) could be observed, while fewer mitochondria became swollen, and vacuolization (up arrows in Figure 6c) compared with the CdSe groups was observed.

Undoubtedly, modified OPC has a protective effect on the lung injury induced by CdSe nanorods and, in particular, has obvious properties of antioxidation and free radical scavenging. The oral administration of modified OPC could significantly prevent pulmonary fibrosis induced by CdSe nanorods in lung tissues. However, whether CdSe nanorods could change the permeability of cells under the protection of modified OPC is not clear. Thus, we employed the A549 cells for the in vitro study. The oxidative stress assessment of A549 cells showed that the activity of T-SOD and T-AOC in A549 cells of the CdSe groups was obviously lower than that of the control groups, while the MDA levels were significantly higher than those in the control groups.

**Figure 7:**
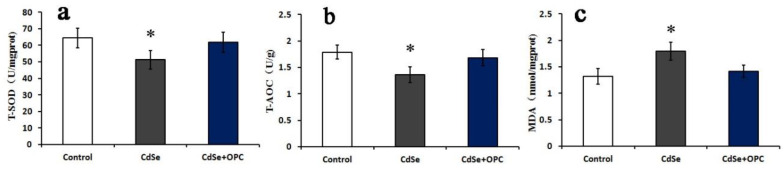
The bar graph showing T-SOD (**a**), T-AOC activity (**b**), and MDA content (**c**) in A549 cells. They were measured using microplate readers represented as mean ± SD. (* *p* < 0.05, as compared with the normal control groups).

The OPC groups had a dramatic difference when compared to the CdSe groups, the activity of T-SOD and T-AOC also slightly decreased, and the MDA levels increased when compared with the control groups (Figure 7). Contracted with lung tissues, the activity of T-SOD and T-AOC and the MDA levels of A549 cells in the OPC group just had a marginal difference with those in the control groups, due to the elimination of free radicals by OPC, a decrease in the excess ROS production, and contraction with the in vivo pathway. OPC is likely to play a more effective role in vitro experiments, since only part of OPC could be absorbed within SD rats through gavage.

**Figure 8:**
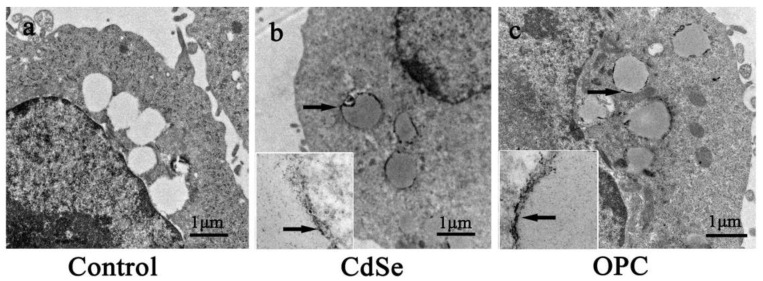
Representative photos of the ultrathin sections made with A549 cells after 24 h treatment. (**a**) the control group, (**b**) 20 μg/mL of CdSe nanorods, and (**c**) 20 μg/mL of CdSe nanorods and 20 μg/mL of OPC. The illustration in (**b**,**c**) are magnified fat droplets, the arrows in (**b**,**c**) are aggregated CdSe nanorods.

The electron microscopic images in the transmission mode showed the appearance of lipid-containing vesicles and nanoparticles internalized in the fat droplet. The three groups did not observe any obvious organelle lesions. The CdSe nanorods mainly aggregate in the fat droplet. The control groups had transparent and clear fat droplets (Figure 8a), while the CdSe and OPC groups had semitransparent fat droplets. CdSe nanorods mainly aggregated at fat droplets (Figure 8b,c), and the two groups had no obvious difference, thus suggesting that OPC could eliminate free radicals, decrease the excess ROS production, but have no effect on the permeability of cells and organelles. 

## 4. Discussion

The biological effects and environmental safety of nanoparticles have attracted widespread attention with the continuous development of nanoparticle technology in recent years [1,3]. When considering the novel type of property of CdSe nanorods and their foreseen widespread application, this led us to investigate their potential adverse health effects at an early stage [44,56]. Various metal selenide nanoparticles have been shown to induce tissue damage and cell death after internalization [40,58]. Some researches considered that metal selenide nanoparticles could dissolve in a culture medium, which causes the release of ions and induces cell death [59].

To observe the evidence of a pathologically damaging impact on the lung tissues of a rat by CdSe nanorods, H&E staining was employed to observe changes in the lung tissue structures of a rat. The space between pulmonary alveoli increased, and the infiltration of inflammatory cells such as macrophages was more obvious in the CdSe nanorod groups, including extensive inflammatory responses such as alveolar walls widening, alveolar mucosa with swelling, congestion as a prelude to the development of lung fibrosis, and lung tissue fiber deposition. Particularly in the late stage for 60 and 90 days, the damage was more serious. However, there was no obvious tissue inflammation and interstitial fiber deposition in the control groups. In addition, we suspect that the 30 mg and 45 mg/lung doses could be construed as lung overload, and thus the responses observed in these animals may be due to failed clearance and not a true inflammatory and fibrotic response to CdSe nanorods.

The Masson’s trichrome staining and hydroxyproline levels were evaluated. Collagen deposition indicates pulmonary fibrosis, and the hydroxyproline levels significantly increased in the SD rats, which intermittently tented CdSe nanorods on days 30 and became more serious on days 60 and 90. The results demonstrated that pulmonary instillation of CdSe nanorods could induce pulmonary fibrosis in a short time. The formation of pulmonary fibrosis is mainly attributed to increased fibrosis factors [60] and anti-fibrosis factor deficiency that cause abnormalities of the extracellular matrix during the pulmonary fibrosis process [61,62]. Shvedova et al. (2009) [63] demonstrated that carbon nanotubes could induce pulmonary fibrosis by oxidation damage. van Berlo et al. (2014) [64] demonstrated that MWCNT induces inflammatory cell influx, markedly increased granuloma formation, and induce clear fibrotic responses. These lesions are characterized by the concentration of one-dimensional nanostructures, higher cellular density, accumulation of immune cells, especially macrophages, and the nodular histiocyte hyperplasia and fibroblastic proliferation increased.

Nanoparticles-mediated tissue damage and cell death are attributed to the production of ROS, leading to oxidative stress caused by nanoparticles with a small size and large surface area to volume ratio [39,65]. T-SOD is an important superoxide dismutase in life systems or biological bodies and is widely distributed in all kinds of living organisms [66]. It also converts harmful superoxide free radicals to hydrogen peroxide in living cells. In the presence of catalase and peroxidase, hydrogen peroxide broke down into harmless water molecules. Hence, it protects and/or recovers cells from free radical damages [67,68]. T-AOC not only accurately reflects the oxidation state but also indirectly evaluates the activity of oxygen free radicals [69]. The increase in free radicals could reduce the body’s T-AOC vigor. MDA is an end-product formed from the peroxide decomposition of unsaturated fatty acids. The content of MDA directly reflects the strength and speed of body lipid peroxidation. The MDA indirectly illustrates the severity of damaged tissues or cells. MDA is often considered an important indicator to estimate the damage induced by oxidative stress [70,71]. The present study shows that CdSe nanorods are capable of producing ROS in lung tissues after pulmonary instillation for 30 days, and the continuous accumulation reveals a time-dependent manner up to 60 and 90 days, CdSe nanorods decreased the vitality of T-SOD and T-AOC in lung tissues of a rat and increased the MDA level and lipid peroxidation products, indicating that the CdSe nanorods induced oxidative damage in the lung tissues of a rat.

The toxicity of CdSe nanoparticles has been demonstrated to be associated with ROS generation, mitochondrial dysfunction, and autophagy-related cell death [72]. The pathogenesis of CdSe nanorod-induced pulmonary fibrosis is probably related to the combined effects of different types of cells, including pneumocytes, inflammatory cells, endothelial cells, fibroblasts, and mitochondrial disruption [73]. In the present study, the electron microscopic images in the transmission mode could show that pulmonary instillation of CdSe nanorods induced mitochondrial cristae broken, and vacuolization. OPC has been reported to possess a variety of potent properties, including antioxidant, anti-inflammation, radical scavenging, renal protection, and antitumor activities [74,75]. Many physiological benefits of OPC have been attributed to its antioxidant as well as free radical scavenging properties. It is also found that OPC could inhibit lipid peroxidation and modulate the activity of regulatory enzymes and has a dramatic scavenging ability towards biochemically generated superoxide anion, hydroxyl, and peroxyl radicals. The oral administration of OPC could significantly prevent those negative changes induced by CdSe nanorods in lung tissues and had a protective effect on CdSe nanorod-induced pulmonary fibrosis. Through the assessment of oxidative stress and malonaldehyde in lung tissues, both in vivo and in vitro experiments, the OPC solution could significantly increase the content of antioxidant enzymes, scavenge free radicals, and reduce the lipid peroxidation. As for the histopathological change of lung tissues, OPC significantly improved inflammatory changes of lung tissues, including slowing down the alveolar walls swelling and alveolar mucosa congestion, improving scores of lung alveolitis and fibrosis, and lowering the hydroxyproline content, which directly correlated with the collagen deposition. The above results suggested that oral administration of OPC could benefit not only the early inflammatory stage but also the later stages of CdSe nanorod-induced pulmonary fibrosis. Once safety accidents caused by inhalation of CdSe nanorods or other one-dimensional nanomaterials happened, oral OPC could be an effective therapy to treat pulmonary fibrosis.

## 5. Conclusions

In conclusion, the present study indicates that one-dimensional CdSe nanorods could induce an extensive inflammatory response, elevate ROS, induce pulmonary fibrosis, and reveal a time accumulation appearance. OPC has a protective effect on lung injury induced by CdSe nanorods, which might be related to its anti-oxidative and anti-inflammatory properties. OPC appears to be an effective therapy against pulmonary fibrosis, although the mechanisms remain and need to be further explored.

## Data Availability

Not applicable.

## Supplementary Materials

The following supporting information can be downloaded at: https://www.mdpi.com/article/10.3390/toxics10110673/s1. Figure S1. Schematic representation of the experiment. Figure S2. Characterization of the synthesized CdSe nanorods. (a) TEM observation; and (b) powder XRD analysis. Table S1. The particle size, hydrodynamic diameter (DLS) and zeta potential of the synthesized CdSe nanorods.
