# Supplementary material for: Pulmonary Fibrosis Induced by CdSe Nanorods and the Therapy with Modified Procyanidinere
Source: Toxics. 2022 Nov 8;10(11):673. doi: 10.3390/toxics10110673 (PMC9693992; doi:10.3390/toxics10110673)
Supplement: Supplementary file 1 [file toxics-10-00673-s001.zip › toxics-2002682-supplementary.pdf]

# Supporting information

## Pulmonary fibrosis induced by CdSe nanorods and the therapy with modified procyanidinere

Zongkai Yue[a,b], Ruiren Zhou[c], Qingzhao Li[d], Shaohu Ouyang[b], Lu Liu[b], Qixing Zhou[b,*]

[*]Correspondence: zhouqx@nankai.edu.cn (Q.Z.)

[a] Laboratory of Environmental Protection in Water Transport Engineering, Tianjin Research Institute for Water Transport Engineering, Ministry of Transport of The People's Republic of China, Tianjin 300456, China

[b] Ministry of Education Key Laboratory of Pollution Processes and Environmental Criteria / Tianjin Key Laboratory of Environmental Remediation and Pollution Control, College of Environmental Science and Engineering, Nankai University, Tianjin300071, China

[c] Department of Biological and Agricultural Engineering, Texas A&M University, College Station, TX 77843-2117, USA

[d] Preventive Medicine Department and Department of Biological Science, Hebei United University, Tangshan 063000, China

## Supplementary Figures

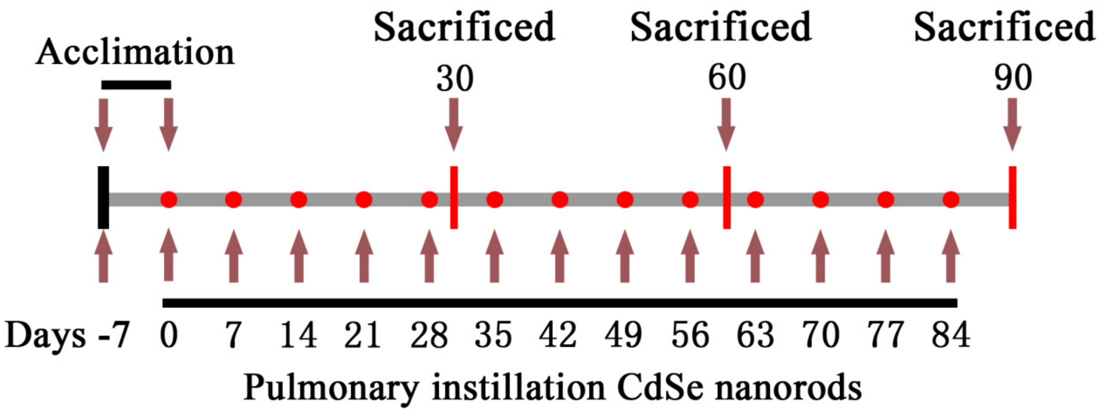


**Figure S1.** Schematic representation of the experiment

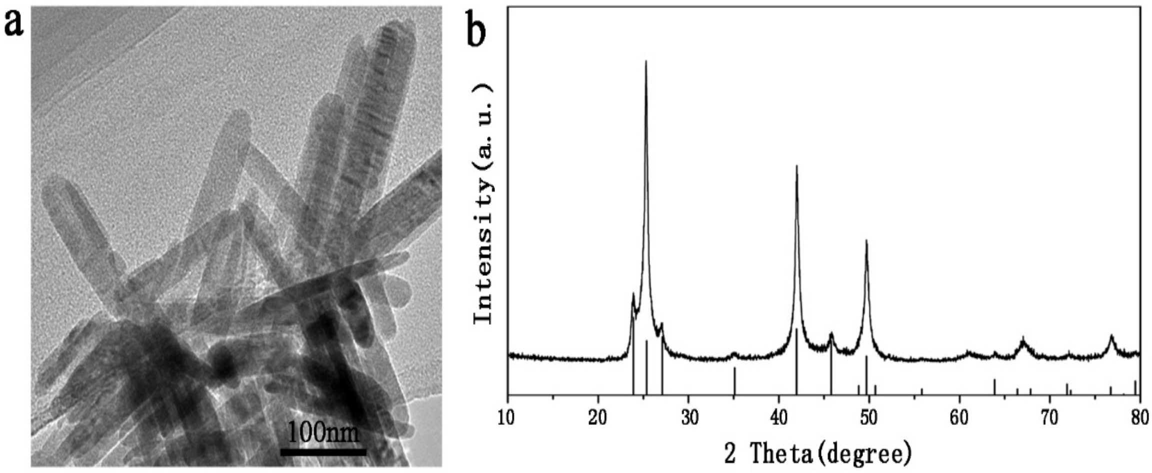


**Figure S2.** Characterization of the synthesized CdSe nanorods. (a) TEM observation; and (b) powder XRD analysis.

## Supplementary Tables

**Table S1.** The particle size, hydrodynamic diameter (DLS) and zeta potential of the synthesized CdSe nanorods.

| **Nanorods** | Diameter(nm) | Length(nm) | DLS (saline, 0h, nm) | DLS (saline, 24h, nm) | DLS (RPMI medium, 0h, nm) | DLS (RPMI medium, 24h, nm) | Zeta potential (mV) |
|---|---|---|---|---|---|---|---|
| **CdSe** | 40-60 | 150-300 | 380 | 595 | 321 | 332 | -18.2±4.06 |
